# Anemia, Blood Transfusion Requirements and Mortality Risk in Human Immunodeficiency Virus-Infected Adults Requiring Acute Medical Admission to Hospital in South Africa

**DOI:** 10.1093/ofid/ofv173

**Published:** 2015-11-12

**Authors:** Andrew D. Kerkhoff, Stephen D. Lawn, Charlotte Schutz, Rosie Burton, Andrew Boulle, Frank J. Cobelens, Graeme Meintjes

**Affiliations:** 1 Department of Medicine, University of California San Francisco School of Medicine; 2 Department of Global Health, Academic Medical Center, Amsterdam Institute for Global Health and Development, University of Amsterdam, The Netherlands; 3 Faculty of Health Sciences, The Desmond Tutu HIV Centre, Institute of Infectious Disease and Molecular Medicine, University of Cape Town, South Africa; 4 Department of Clinical Research, Faculty of Infectious and Tropical Diseases, London School of Hygiene and Tropical Medicine, United Kingdom; 5 Department of Medicine, Faculty of Health Sciences, University of Cape Town; 6 Clinical Infectious Diseases Research Initiative, Institute of Infectious Disease and Molecular Medicine, University of Cape Town; 7 Khayelitsha District Hospital, Cape Town; 8 Faculty of Health Sciences, Centre for Infectious Disease Epidemiology and Research, School of Public Health and Family Medicine, University of Cape Town; 9 Department of Health, Provincial Government of the Western Cape, South Africa; 10 KNCV Tuberculosis Foundation, The Hague, The Netherlands; 11 Department of Medicine, Imperial College, London, United Kingdom

**Keywords:** Africa, AIDS, anemia, blood transfusion, HIV, hospitalized, mortality

## Abstract

***Background.*** Morbidity and mortality remain high among hospitalized patients infected with human immunodeficiency virus (HIV) in sub-Saharan Africa despite widespread availability of antiretroviral therapy. Severe anemia is likely one important driver, and some evidence suggests that blood transfusions may accelerate HIV progression and paradoxically increase short-term mortality. We investigated the relationship between anemia, blood transfusions, and mortality in a South African district hospital.

***Methods.*** Unselected consecutive HIV-infected adults requiring acute medical admission to a Cape Town township district hospital were recruited. Admission hemoglobin concentrations were used to classify anemia severity according to World Health Organization/AIDS Clinical Trials Group criteria. Vital status was determined at 90 days, and Cox regression analyses were used to determine independent predictors of mortality.

***Results.*** Of 585 HIV-infected patients enrolled, 578 (98.8%) were included in the analysis. Anemia was detected in 84.8% of patients and was severe (hemoglobin, 6.5–7.9 g/dL) or life-threatening (hemoglobin, <6.5 g/dL) in 17.3% and 13.3%, respectively. Within 90 days of the date of admission, 13.5% (n = 78) patients received at least 1 blood transfusion with red cell concentrate and 77 (13.3%) patients died. In univariable analysis, baseline hemoglobin and receipt of blood transfusion were associated with increased mortality risk. However, in multivariable analysis, neither hemoglobin nor receipt of a blood transfusion were independently associated with greater mortality risk. Acquired immune deficiency syndrome-defining illnesses other than tuberculosis and impaired renal function independently predicted mortality.

***Conclusions.*** Newly admitted HIV-infected adults had a high prevalence of severe or life-threatening anemia and blood transfusions were frequently required. However, after adjustment for confounders, blood transfusions did not confer an increased mortality risk.

Despite the unprecedented scale-up of antiretroviral therapy (ART) in sub-Saharan Africa over the last decade, morbidity and mortality among hospitalized inpatient persons infected with human immunodeficiency virus (HIV) remain very high [1, 2]. Anemia is the most common hematological complication of HIV disease, it is highly prevalent among persons living with HIV/acquired immune deficiency syndrome (AIDS) in sub-Saharan Africa [3–6], and it may be an important driver of morbidity and mortality among inpatients infected with HIV. HIV-related anemia is associated with fatigue, decreased quality of life [7], and a greatly increased mortality risk [8–11]. Although the South African HIV Clinician's Society has published recommendations on the management of anemia and blood transfusions among patients infected with HIV, there are no normative international guidelines for the management of HIV-related anemia in sub-Saharan Africa or other resource-limited settings [12].

ART is associated with substantial hemoglobin recovery, resulting in resolution of anemia in a majority of HIV-infected African patients without additional specific hematological interventions [3, 5, 6, 13, 14]. ART is thus the key therapeutic intervention for HIV-related chronic anemia, but weeks or months may be required for adequate hemoglobin recovery to occur with ART. However, patients may also present acutely to healthcare settings with severe or life-threatening anemia, requiring immediate correction. This may include administration of blood transfusions when considered clinically appropriate. Although it may be expected that HIV-infected hospitalized patients in sub-Saharan Africa have high transfusion requirements, few data have been published data on this topic [15–17]. In addition, some evidence suggests that blood transfusions are associated with modest increases in plasma HIV-1 RNA levels and increased mortality risk [11, 18–20]. This has led some clinicians to conclude that transfusions should be used sparingly among patients infected with HIV [12]. The limited evidence to date to support such caution is derived exclusively from North America, and thus this issue has not yet been investigated in sub-Saharan Africa, where the prevalence of HIV-related anemia remains high. Therefore, we sought to determine whether blood transfusions were independently associated with an increased mortality risk among HIV-infected adults requiring acute medical admission to a district-level hospital in Cape Town, South Africa.

## METHODS

GF Jooste Hospital served as the public sector adult referral hospital for a community of approximately 1.3 million people at the time of the study. Patients seen at public sector primary care, HIV, or tuberculosis (TB) clinics or by private general practitioners could be referred to the hospital. Those ill enough to require admission were admitted to the medical ward. The local antenatal HIV seroprevalence is approximately 35%, and the large burden of TB is well described [21, 22]. The Research Ethics Committees of the University of Cape Town and the London School of Hygiene & Tropical Medicine approved this study, and all patients provided written informed consent before participation.

This study was nested within a prospective cohort study in which the parent study sought to evaluate new approaches to the rapid diagnosis of HIV-associated TB [23]. HIV-infected persons ≥18 years of age requiring acute admission to a medical ward were eligible for inclusion. Patients were prospectively recruited 4 days per week from June 2012 to October 2013. Any patient with an unknown or previously negative test was offered HIV testing. A positive HIV serostatus was confirmed in parallel by 2 rapid assays.

### Procedures

Upon enrollment, demographic and clinical details were recorded for all patients. Patients were systematically investigated for TB by obtaining a combination of respiratory and nonrespiratory clinical specimens as previously described [23]. A venous blood sample was collected for the measurement of C-reactive protein (CRP) and HIV viral load in keeping with the protocol of the parent study. Hemoglobin concentrations were measured using the ADVIA 2120 hematology analyzer (Siemens Healthcare Diagnostics, Erlangen, Germany).

### Patient Outcomes and Use of Blood Products

Mortality and loss to follow-up (LTFU) within 90 days after study entry was determined using patient case notes and the medical ward register in addition to 6 regional and national electronic health record databases [24]. All data sources were cross-referenced for consistency, and any discrepancies in patient outcomes were resolved.

The decision to administer a blood transfusion was made by the clinical team responsible for the patient, and there was no hospital guideline defining a recommended hemoglobin “threshold” level for blood transfusion. Electronic records from the local Red Cross Hospital blood bank were searched to identify patients receiving a blood transfusion with red blood cell concentrate (RCC) between 6 months before study enrollment to 6 months after study enrollment (12-month interval). However, because transfusions administered between 7 days prior to and 90 days after the study admission date were most likely to be associated with mortality, only blood transfusion data from this time interval was utilized. Costs associated with blood products and transfusion services were also extracted from billing invoices from the Western Province blood transfusion service. Costs were estimated “top-down” and included fees associated with blood products, screening and testing, service fees, and associated value-added tax; fees associated with clinician services and medical supplies necessary for transfusions were not included.

### Data Analysis and Definitions

Analyses were restricted to data from patients with hemoglobin levels available from the time of hospital admission. Admission hemoglobin levels as well as full blood counts, blood chemistry (including creatinine), and the results of all mycobacterial and other microbiological investigations were extracted from the National Health Laboratory Service computerized data system. Anemia severity was classified into mutually exclusive groups using admission hemoglobin levels according to a combination of World Health Organization [25] and AIDS Clinical Trials Group (ACTG) [26] criteria. These are as follows: no anemia (≥13.0 g/dL for men, ≥12.0 g/dL for females), mild anemia (11.0–12.9 g/dL for men, 11.0–11.9 g/dL for females), moderate anemia (8.0–10.9 g/dL for males and females), severe anemia (6.5–7.9 g/dL for males and females), and life-threatening anemia (<6.5 g/dL for males and females). Patient case notes and laboratory results were reviewed by 2 investigators (G.M. and A.D.K.) and were used to classify patients into 5 mutually exclusive groups based upon a primary clinical diagnosis: newly diagnosed TB, clinical deterioration of known TB disease, AIDS-defining illness (other than TB), major organ dysfunction/noncommunicable disease, or “other,” as previously described [24]. “Cardiopulmonary illness” was defined by any patient for whom their primary admission diagnosis was related to a cardiac and/or pulmonary disease, including newly diagnosed or worsening TB disease. Creatinine levels upon admission were used to calculate an estimated glomerular filtration rate (eGFR) [27]. For multivariable analyses, a patient was considered to have received a blood transfusion if they were transfused with RCC at any point between 7 days before and 90 days after study entry.

All data analyses were conducted using Stata version 12.0 (College Station, TX). Proportions were compared using either Fisher's exact tests or χ^2^ tests, and medians were compared using either Wilcoxon rank-sum tests or Kruskal–Wallis tests. Multivariable logistic regression analyses were undertaken to identify variables associated with receipt of a blood transfusion. Cox proportional-hazards regression analyses were performed to identify independent predictors of mortality in the first 90 days after study enrollment and specifically to test whether blood transfusions were independently associated with an increased mortality risk. Person-time was accrued from the date of hospital admission until death, LTFU, or censorship of data at 90 days after hospital admission. Two multivariable models were constructed that included receipt of blood transfusions coded as a binary (yes/no), an ordinal (none, 1–3 [low], 4–7 [medium], ≥8 [high]), or a continuous variable (for each unit increase). The first model, “clinical model”, included only prespecified factors most likely to influence a physician to order a blood transfusion (blood hemoglobin level, a cardiopulmonary illness, and renal function). The second model, “fully adjusted,” included prespecified factors (ie, blood hemoglobin level, clinical diagnosis, confirmed mycobacteremia, renal function) and any covariates in the univariable models meeting a predetermined cutoff of *P* < .1. Likelihood ratio tests were used to investigate statistical hypotheses and interactions between variables. All statistical tests were 2-sided at α = 0.05.

## RESULTS

### Study Profile

Of 609 patients with confirmed HIV infection, 585 were enrolled, and, of these, 578 (98.8%) had admission hemoglobin levels available and were included in this analysis. Patients were typically young, and the majority were female (Table 1). Approximately 85% of patients were aware of their positive HIV-status on admission, but 36% had not yet commenced ART, and viral load was suppressed in only 31% of all patients. Advanced immunodeficiency was common (median CD4 count, 133 cells/µL), and approximately one half of patients had previously been treated for TB disease (Table 1).

**Table 1.** Baseline Characteristics by WHO/ACTG Anemia Severity Classification (All Values Are Numbers [%] Unless Otherwise Stated)

Patient characteristics	All Patients, n = 578 (100%)	No Anemia, n = 88 (15.2%)	Mild Anemia, n = 91 (15.7%)	Moderate Anemia, n = 222 (38.4%)	Severe Anemia, n = 100 (17.3%)	Life-threatening Anemia, n = 77 (13.3%)	*P* Value
Age, median (IQR)	35.4 (28.9–41.4)	37.4 (30.3–45.2)	38.0 (31.2–44.3)	34.8 (29.1–41.5)	32.9 (27.6–39.2)	32.8 (27.3–38.5)	<.001
Female	333 (57.6)	49 (55.7)	42 (46.2)	137 (61.7)	61 (61.0)	44 (57.1)	.134
HIV newly diagnosed	91 (15.7)	13 (14.8)	10 (11.0)	27 (12.2)	22 (22.0)	19 (24.7)	.023
ART status							
ART-naive	206 (35.6)	35 (39.8)	28 (30.8)	66 (29.7)	43 (43.0)	34 (44.2)	.069
Current ART use	260 (45.0)	41 (46.6)	47 (51.7)	107 (48.2)	34 (34.0)	31 (40.3)	
ART interrupted	112 (19.4)	12 (13.6)	16 (17.6)	49 (22.1)	23 (23.0)	12 (15.6)	
Patients currently receiving ART**^a^**							
Treatment duration (years), median (IQR)	1.0 (0.2–2.7)	1.8 (0.9–3.9)	0.7 (0.2–2.8)	0.7 (0.2–2.5)	0.9 (0.2–2.8)	0.7 (0.2–2.2)	.081
ART for <90 d	67 (26.8)	4 (10.3)	10 (22.7)	32 (31.1)	13 (38.2)	8 (26.7)	.048
AZT-containing regimen	37 (10.0)	7 (13.2)	6 (9.5)	18 (11.5)	3 (5.3)	3 (6.0)	.574
Hematological tests, median (IQR)							
Hemoglobin (g/dL)	9.5 (7.6–11.3)	13.6 (12.8–14.3)	11.5 (11.2–11.8)	9.5 (8.8–10.1)	7.5 (6.9–7.7)	5.4 (4.6–6.0)	<.001
MCV (fL)^b^	89 (83–95)	94 (88–99)	91 (86–97)	89 (84–94)	84 (79–91)	85 (79–92)	<.001
MCHC (g/dL)^c^	32.9 (32.1–33.7)	33.5 (32.9–34.2)	33.3 (32.6–34.0)	32.8 (32.1–33.6)	32.7 (31.6–33.3)	32.1 (30.9–32.8)	<.001
RDW (%)^c^	14.5 (12.9–16.3)	13.1 (12.5–14.3)	13.4 (12.5–15.0)	14.6 (13.2–16.1)	15.3 (14.1–17.3)	16.3 (13.7–18.5)	<.001
White cell count (×10^9^ cells)^d^	6.9 (4.6–10.1)	6.4 (4.6–10.2)	7.3 (5.1–11.1)	7.0 (5.1–10.2)	6.5 (3.8–9.2)	7.0 (4.0–10.0)	.109
Platelets (×10^9^ cells)^b^	260 (188–355)	244 (178–309)	270 (206–397)	280 (195–356)	235 (175–338)	237 (140–383)	.022
CD4 cell count (cells/μL), median (IQR)^e^	133 (53–268)	239 (111–459)	200 (106–370)	129 (53–235)	72 (33–179)	71 (32–190)	<.001
Log viral load (copies/mL), median (IQR)^f^	4.2 (1.6–5.5)	3.6 (1.6–5.0)	3.7 (1.6–5.2)	4.0 (1.6–5.5)	5.4 (3.2–5.9)	4.2 (2.3–5.4)	<.001
Viral suppression (viral load<400 copies/mL)	174 (31.0)	34 (39.1)	33 (37.1)	67 (30.6)	17 (18.3)	23 (31.1)	.024
CRP (mg/L), median (IQR)^g^	71 (25–138)	21 (7–102)	59 (23–119)	73 (28–147)	100 (50–169)	90 (53–142)	<.001
Creatinine (μmol/L), median (IQR)^h^	68 (52–93)	70 (54–84)	66 (53–83)	65 (51–84)	67 (52–109)	85 (52–194)	.028
eGFR classification (mL/min/1.73 m^2^)^h^							
<30	39 (6.8)	3 (3.5)	0	11 (5.0)	9 (9.0)	16 (20.8)	<.001
≥30	538 (93.2)	84 (96.6)	91 (100)	211 (95.1)	91 (91.0)	61 (79.2)	
Tuberculosis							
Positive WHO symptom screen^e^	537 (93.2)	74 (84.1)	83 (91.2)	208 (94.6)	98 (98.0)	74 (96.1)	.003
History of previous TB^e^	259 (45.0)	37 (42.1)	37 (40.7)	107 (48.6)	50 (50.0)	28 (36.4)	.239
Positive mycobacterial blood culture^i^	45 (8.2)	1 (1.2)	1 (1.2)	14 (6.7)	17 (17.5)	12 (16.2)	<.001

Abbreviations: ART, antiretroviral therapy; AZT, zidovudine; CRP, C-reactive protein; eGFR, estimated glomerular filtration rate; fL, femtoliters; HIV, human immunodeficiency virus; IQR, interquartile range; MCV, mean corpuscular volume; MCHC, mean corpuscular hemoglobin concentration; RDW, red cell distribution width; TB, tuberculosis; WHO, World Health Organization.

^a^ n = 260; ^b^ n = 564; ^c^ n = 563; ^d^ n = 575; ^e^ n = 576; ^f^ n = 562; ^g^ n = 554; ^h^ n = 577; ^i^ n = 552.

### Anemia Diagnoses

The median hemoglobin level was 9.5 g/dL, and 84.8% (n = 490) of all patients were anemic. Anemia was classified as mild, moderate, severe, and life-threatening in 15.7% (n = 91), 38.4% (n = 222), 17.3% (n = 100), and 13.3% (n = 77) of 578 patients, respectively (Table 1). Greater anemia severity was associated with younger age, a new HIV diagnosis, and having started ART within the previous 90 days (Table 1). Those with more severe anemia were also more likely to have more advanced immunodeficiency, higher HIV viral loads, higher CRP levels, and lower eGFRs (Table 1). Lower mean corpuscular values and mean corpuscular hemoglobin values as well as increased red cell distribution widths were also associated with greater severity of anemia. In addition, those with greater anemia severity were more likely to have symptoms suggestive of TB and also have mycobacteremia (Table 1). Only 37 (6.4%) of all patients were receiving a zidovudine (AZT)-containing regimen, and AZT use was not associated with anemia severity.

### Clinical Diagnoses Among Patients With Anemia

Patients were classified into 1 of 5 mutually exclusive groups based on their primary diagnosis leading to hospital admission (Table 2). Newly diagnosed TB disease was the most common diagnosis among the overall cohort and accounted for almost half of all diagnoses among those with severe or life-threatening anemia. Approximately one quarter of patients with life-threatening anemia had major organ dysfunction or a noncommunicable disease. Acquired immune deficiency syndrome-defining illnesses other than TB did not constitute a large proportion of those with more severe anemia.

**Table 2.** Overview of Primary Clinical Diagnoses^a^ by WHO/ACTG Anemia Severity Classification

Primary clinical diagnoses	All Patients (n = 575)^b^	No Anemia (n = 87)	Mild Anemia (n = 91)	Moderate Anemia (n = 221)	Severe Anemia (n = 99)	Life-Threatening Anemia (n = 77)
Newly diagnosed TB	196 (34.1)	22 (25.3)	23 (25.3)	71 (32.1)	52 (52.5)	28 (36.4)
Clinical deterioration of known TB	42 (7.3)	3 (3.5)	5 (5.5)	20 (9.1)	8 (8.1)	6 (7.8)
AIDS-defining illness other than TB	64 (11.1)	9 (10.3)	13 (14.3)	28 (12.7)	8 (8.1)	6 (7.8)
Major organ dysfunction or noncommunicable disease	80 (13.9)	18 (20.7)	13 (14.3)	22 (10.0)	9 (9.1)	18 (23.4)
Other diagnosis	193 (33.6)	35 (40.2)	37 (40.7)	80 (36.2)	22 (22.2)	19 (24.7)

Abbreviations: AIDS, acquired immune deficiency syndrome; TB, tuberculosis; TB-IRIS, TB-associated immune reconstitution inflammatory syndrome; WHO, World Health Organization.

^a^ Patients were categorized into a primary clinical diagnosis according to the following methodology: (1) new TB diagnosis (took priority over other diagnoses); (2) clinical deterioration of TB cases during treatment (included the following underlying causes: poor adherence, drug-resistant TB, TB-IRIS, hemoptysis, and pneumothorax); (3) AIDS-defining illnesses other than TB (included: opportunistic infections and AIDS-related malignancies); (4) noncommunicable diseases (included: non-AIDS cancer, diabetes, hypertensive complications, heart failure, and asthma) or major organ dysfunction not attributable to one of the above categories (included: renal and liver failure, bone marrow dysfunction, seizures, and stroke). “Other diagnoses” included the following: other bacterial infection (including pneumonia and dysentery) venous thromboembolism, drug-related (adverse effects or overdose), psychiatric illness, or other unclassifiable diagnoses.

^b^ Three patients could not have a primary clinical diagnosis made based on available information.

### Blood Transfusion Requirements and Associated Costs Among All Patients

Within 90 days after hospital admission and study enrollment, 13.5% (n = 78) of all patients and 15.7% (n = 77) of patients with anemia of any severity at the time of hospital admission received at least 1 blood transfusion with RCC; 61.0% (n = 47) of patients with life-threatening anemia at admission and 16.0% (n = 16) of those with severe anemia at admission were transfused. A total of 246 units were transfused, and the mean number of units per patient was 3.2 (standard deviation = 2.3; range, 1–12); 42.3% (n = 33) of those requiring at least 1 unit of RCC had multiple blood transfusions within 90 days. The cost of RCC alone (n = 246 units) in those requiring a blood transfusion was 192 950 South African Rand (ZAR [$19 523 US dollars at study end date]). When service fees and taxes were included, the total overall cost was 256 370 ZAR ($25 940), and thus the mean cost per patient requiring transfusion was 3287 ZAR ($333).

### Relationship Between Blood Transfusions and Mortality

Overall, 77 (13.3%) of patients died within 90 days, of which 68 (88.3%) had some degree of anemia; 16.9% (n = 13) had severe and 24.7% (n = 19) had life-threatening anemia, respectively. Of patients receiving a blood transfusion, 26.0% (n = 20) died compared with 11.6% (n = 58) of patients who were not transfused (*P* < .001). The 20 patients who died despite receiving a transfusion typically had advanced immunodeficiency (median admission CD4 count, 68 cells/µL) and very severe anemia (median hemoglobin level, 6.0 g/dL). In addition, a large proportion had TB disease and additional significant comorbidities (Supplementary Table 1).

**Figure 1.**
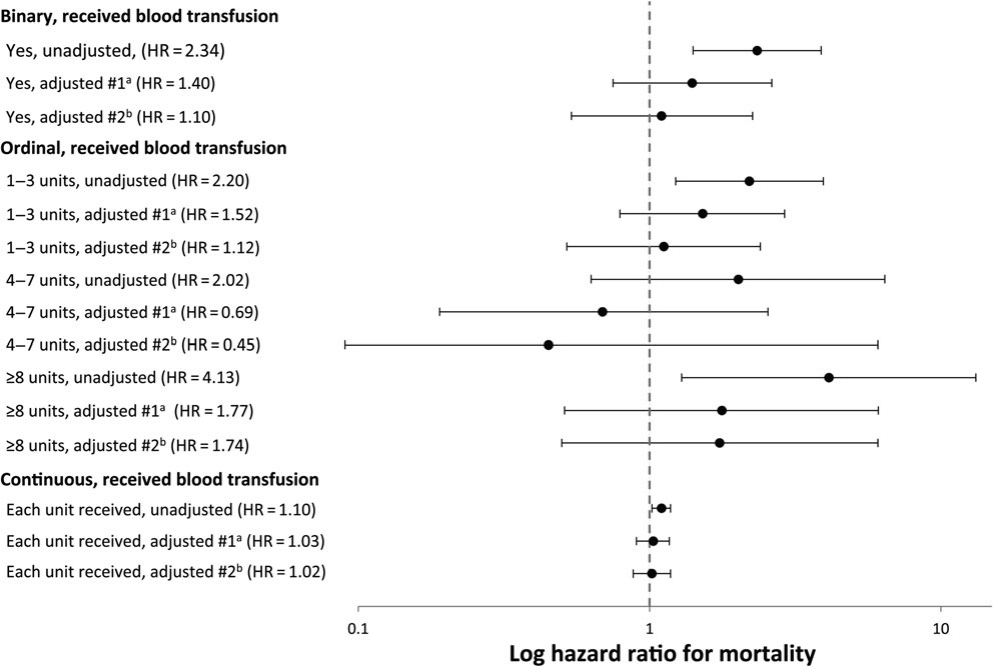
Adjusted hazard ratios and associated 95% confidence intervals for mortality stratified by blood transfusion status. ^a^Model #1 (clinical model) adjusted for hemoglobin concentration, cardiopulmonary illness status, and renal function (estimated glomerular filtration rate <30 mL/min/1.73 m^2^; yes or no). ^b^Model #2 (fully adjusted model) adjusted for age, gender, CD4 cell count, hemoglobin concentration, C-reactive protein concentration, renal function (eGFR <30; yes or no), mycobacterial blood culture result, and primary clinical diagnosis category. The comparator group for all analyses was those who did not receive a blood transfusion.

In univariable Cox regression analyses, receipt of blood transfusion was associated with increased mortality risk, irrespective of whether it was coded as a binomial, ordinal, or continuous variable (Table 3). However, regardless of how receipt of blood transfusions were coded, in multivariable analyses neither model demonstrated evidence that blood transfusions were associated with a greater risk of mortality (Figure 1). Having an eGFR < 30 (consistent with chronic renal disease) was independently associated with mortality in all multivariable models. Patients with a cardiopulmonary illness or those who were diagnosed with either an AIDS-defining illness or noncommunicable disease or major organ dysfunction also had an independently higher mortality risk. Lower hemoglobin levels were independently associated with poor survival in some but not all multivariable models (Table 1 and Supplementary Table 2).

**Table 3.** Cox Multivariable Analysis for Risk Factors Associated With Mortality (Blood Transfusion Coded as a Binary Variable)

	Unadjusted HR (95% CI)	*P* Value	“Clinical Model” Adjusted HR (95% CI)	*P* Value	“Classic Model” Adjusted HR (95% CI)	*P* Value
Age, for each year increase	1.00 (.98–1.02)	.961			1.00 (.98–1.03)	.834
Male	1.02 (.65–1.60)	.936			0.81 (.48–1.36)	.415
ART status						
Current use	1.0	.919				
Naive	1.02 (.62–1.69)					
Defaulted	1.13 (.62–2.05)					
Previous history of TB treatment						
No	1.0	.800				
Yes	0.94 (.60–1.48)					
History of shortness of breath						
No	1.0	.733				
Yes	1.08 (.69–1.69)					
CD4 (cells/μL), for every 50-unit decrease	1.10 (1.02–1.19)	.004			1.06 (.97–1.15)	.186
Viral load (copies/mL), for each log-unit increase	0.99 (.87–1.13)	.871				
Hemoglobin (g/dL), for each unit decrease	1.15 (1.06–1.25)	.001	1.08 (.98–1.19)	.096	1.09 (.98–1.22)	.107
CRP (mg/L), for each 10-unit increase	1.03 (1.01–1.06)	.007			1.03 (1.00–1.06)	.050
eGFR category (mL/min/1.73 m^2^)						
eGFR ≥30	1.0	<.001	1.0	.001	1.0	.010
eGFR<30	3.72 (2.08–6.64)		3.04 (1.65–5.59)		2.54 (1.31–4.93)	
Confirmed mycobacteremia						
No	1.0	.102			1.0	.158
Yes	1.82 (.94–3.53)				1.97 (.78–4.93)	
Clinical Diagnosis						
“Other”	1.0	.002			1.0	<.001
New TB	1.10 (.59–2.05)				0.68 (.30–1.52)	
Deterioration of TB	1.00 (.34–2.94)				1.03 (.33–3.21)	
AIDS-defining illness (other than TB)	3.40 (1.80–6.43)				3.40 (1.62–7.16)	
NCD/MOD	1.84 (.92–3.67)				2.33 (1.05–5.14)	
Cardiopulmonary illness						
Yes	1.0	.050	1.0	.027		
No	1.57 (1.00–2.49)		1.67 (1.05–2.64)			
Received a blood transfusion						
No	1.0	.002	1.0	.296^a^	1.0	.792^a^
Yes	2.34 (1.41–3.89)		1.40 (.75–2.63)		1.10 (.54–2.26)	

Abbreviations: ART, antiretroviral therapy; CI, confidence interval; CRP, C-reactive protein; eGFR, estimated glomerular filtration rate; HR, hazards ratio; MOD, major organ dysfunction; NCD, noncommunicable disease; TB, tuberculosis.

^a^ There was no evidence for interaction between receipt of blood transfusion and duration of follow-up (time).

### Factors Associated With Blood Transfusion in Patients With Severe Anemia

The median hemoglobin at admission in patients receiving a blood transfusion (n = 78) was 5.9 g/dL. The characteristics of patients who received a transfusion were compared with those of patients who did not; however, to improve clinical relevance, analysis was restricted to those with severe or life-threatening anemia (n = 177). Patients receiving a transfusion were more likely (1) to have higher viral loads, (2) to have been previously treated for TB, (3) to have mycobacteremia, and (4) to have an admission diagnosis related to major organ dysfunction or a noncommunicable disease (Supplementary Table 3). In multivariable models, only lower hemoglobin levels predicted receipt of blood transfusion (Supplementary Table 4).

## DISCUSSION

The large majority of HIV-infected adults requiring acute medical admission to a South African district hospital were anemic, and many had either severe or life-threatening anemia. A substantial proportion of such patients received at least 1 blood transfusion, and, cumulatively, this cohort required the administration of a large number of blood products. In contrast to earlier reports from high-income settings, receipt of a blood transfusion was not associated with an increased risk of short-term mortality after adjusting for potential confounding variables.

Although patients requiring a blood transfusion were 2-fold more likely to die in the first 90 days, after adjusting for confounding variables, receipt of blood transfusion was not independently associated with increased mortality. This finding is in contrast with 3 previous reports from the United States showing that short-term mortality risk was increased among HIV-infected patients requiring transfusion [11, 18, 19]. Several theories have been proposed to explain this relationship, including that blood transfusions may cause transient immunosuppression (transfusion immunomodulation), although this is controversial [28]. Although one study found modestly increased viral load among transfused HIV-infected patients, the clinical significance of the small increase in viral load was not determined [20]. In previous studies, it was thought that leukocytes in blood products may (1) directly activate HIV cellular reservoirs, (2) lead to increased proviral transcription, (3) cause immunosuppression, and (4) be associated with greater mortality. However, this was largely disproved by a randomized trial that demonstrated similar HIV-viral loads and survival probabilities among HIV-infected patients receiving nonleukocyte-reduced blood transfusions compared with those receiving leukocyte-reduced blood transfusions [29]. It has also been suggested that blood transfusions may increase mortality by causing iron overload. Although iron excess is associated with increased mortality in sub-Saharan Africa [30], this relationship has not yet been conclusively proven. Finally, transfusion-associated circulatory overload (TACO) may be associated with mortality in HIV-infected patients with chronic severe anemia (especially those with underlying cardiopulmonary disease), but it is a relatively rare clinical phenomenon [12].

We and others suspect that the relationship between receipt of blood transfusion and increased mortality risk reported in previous studies more or less reflects an inherently higher mortality risk among patients with advanced HIV who are likely to be transfused compared with those who are not. In this way, receipt of a blood transfusion serves as a marker of overall disease severity and mortality risk. Therefore, decreased survival may simply represent confounding by indication [31]. In our study, 20 patients died after receiving a transfusion, and such patients were extremely ill, with advanced HIV disease, often had disseminated TB disease, and most had several concomitant copathologies. We carefully considered covariates possibly confounding the relationship between blood transfusions and mortality and adjusted for them accordingly. Even after doing so, there was no evidence to suggest that an independent association existed between blood transfusion and greater mortality, regardless of whether receipt of a transfusion was coded as a binomial, ordinal, or continuous variable. Previous studies in which the relationship between transfusion and poor survival persisted despite adjusting for potential confounders may also have had residual confounding that is present to some degree in all observational studies [31]. This possibility is partially supported by the finding that an independent relationship between blood transfusions and higher mortality among AIDS patients in New York did not persist after further adjusting for illness severity [18]. If present in our study, residual confounding is probably accounted for by unrecorded markers of disease severity, and if a further adjustment were possible, it would likely be in favor of finding transfusions to not adversely affect prognosis. Unmeasured confounders may possibly even mask a clinical benefit associated with receipt of blood transfusions.

In this patient population, which included patients who were ART-naive and those receiving ART, 85% had anemia, and the prevalence of severe or life-threatening anemia was more than 30%. Previous studies in sub-Saharan Africa have also reported a similarly high prevalence of HIV-related anemia, sometimes approaching as high as 90% [3, 5, 6, 32]; however, such estimates are largely derived among ART-naive, ambulatory outpatients. The high prevalence of severe or life-threatening anemia observed in this study was in part due to the inclusion of patients ill enough to seek acute medical attention, as well as advanced HIV/AIDS disease, and a high rate of opportunistic infections, notably TB. Newly diagnosed TB was the primary diagnosis for more than one third of all patients and accounted for nearly one half of diagnoses among patients with severe or life-threatening anemia. These results are entirely consistent with a study from Malawi in which 43% of hospitalized HIV-infected patients with severe anemia had TB [33] and provide further evidence to suggest that anemia might have high predictive value for HIV-associated TB in high-incidence settings [32, 34, 35]. In addition, patients in our study often had advanced HIV illness with opportunistic infection or major organ dysfunction. Thus, malignancies, nutritional deficiencies, and drug toxicities may all have further contributed to the high observed prevalence and severity of anemia [36].

The large number of blood transfusions received by hospitalized, HIV-infected adults in the present study builds upon a previous report from South Africa which demonstrated that HIV-infected inpatients have higher transfusion requirements than HIV-uninfected patients from the same hospital setting and that such patients utilized a large number of blood products [16]. In the present study, despite a large proportion of patients being transfused, it is unclear whether patients had adequate transfusion support because some patients with life-threatening anemia were not transfused, and many of those who were transfused received only 1–2 units of RCC. This may reflect several factors, including patients presenting late to medical care in extremis and dying before transfusion and also that physicians often prescribe blood transfusions based on a patient's composite clinical picture and not hemoglobin level alone. There was not a standardized institutional protocol for blood transfusions; thus, some of these discrepancies are partially influenced by variability in transfusion practice between doctors, departments, and hospitals. In addition, a perception by local physicians of a shortage in supplies of blood products and their high cost may have resulted in blood products being used sparingly by some.

In many clinical settings in sub-Saharan Africa, there is a shortage of “safe” blood products [37]. Although South Africa has greater healthcare resources than most countries of sub-Saharan Africa, inadequate quantities of blood is often still a reality [38]. In this cohort, blood transfusions for 78 patients accrued a cost of more than $25 000, which is likely an underestimate because this estimate did not include associated private clinician's fees and associated medical supplies, and thus transfusions are associated with substantially increased overall healthcare expenditure [39]. In view of these facts, improved solutions are needed to not only increase the availability of safe and affordable blood products but also to reduce unnecessary blood transfusions and to reduce requirements for blood products [37]. In this study, neither current ART use nor viral suppression was associated with a decreased likelihood of blood transfusion. However, patients requiring hospitalization despite ART use and viral suppression are a minority and are not likely representative of those receiving ART in the larger community. Antiretroviral therapy is strongly associated with the resolution of anemia in a majority of patients [3, 5, 6, 13, 14], and a previous study found that ART was associated with decreased blood transfusion requirements [40]. Thus, increased ART coverage may contribute towards decreased patient morbidity, the increased availability of blood products, and decreased healthcare costs. Prescription of blood transfusions should be based on best practice guidelines where available [12], and further studies are needed to evaluate the impact of ART on blood product use (especially large ART cohort studies), as well as additional strategies for reducing blood transfusion requirements among both HIV-infected and uninfected patients in sub-Saharan Africa [15, 41].

Strengths of this study include that this was a well characterized, prospectively recruited cohort that enrolled all HIV-infected hospitalized adults requiring acute medical admission regardless of reason for admission. An additional strength was a high ascertainment rate of vital status with <6% LTFU, despite lack of routine follow-up in outpatient care. Nevertheless, mortality was possibly underestimated because a proportion of patients LTFU may have died. We comprehensively searched for transfusion requirements; however, the proportion of patients transfused and their corresponding blood product use was likely underestimated as patients occasionally report different names or identifying information and may have presented to healthcare settings outside of the study hospital. This study was conducted at a single site, and there was a relatively small number of transfusions and patient deaths. It should also be noted that although the lack of an independent association between blood transfusions and mortality is likely to be generalizable to other inpatient settings in sub-Saharan Africa, the proportion of patients receiving blood transfusions and the associated use of blood products represent point estimates and will undoubtedly vary across different settings for a number of reasons, including variability in blood transfusion prescription practices. Therefore, the transfusion requirements among patients in this study should not be extrapolated to other settings.

## CONCLUSIONS

More than 30% of HIV-infected hospitalized patients had severe or life-threatening anemia, which resulted in a high proportion of patients requiring a blood transfusion. Receipt of a blood transfusion was not independently associated with greater mortality risk. Thus, we found no evidence to support concerns regarding the potential adverse impact of the administration of blood transfusions to HIV-infected adults in sub-Saharan Africa.

## Supplementary Material

Supplementary Data
