# Supplementary material for: Anemia, Blood Transfusion Requirements and Mortality Risk in Human Immunodeficiency Virus-Infected Adults Requiring Acute Medical Admission to Hospital in South Africa
Source: Open Forum Infect Dis. 2015 Nov 12;2(4):ofv173. doi: 10.1093/ofid/ofv173 (PMC4693115; doi:10.1093/ofid/ofv173)
Supplement: Supplementary Data [file supp_2_4_ofv173__index.html]

Anemia, Blood Transfusion Requirements and Mortality Risk in Human Immunodeficiency Virus-Infected Adults Requiring Acute Medical Admission to Hospital in South Africa — Supplementary Data 

# Anemia, Blood Transfusion Requirements and Mortality Risk in Human Immunodeficiency Virus-Infected Adults Requiring Acute Medical Admission to Hospital in South Africa

## Supplementary Data

Supplementary Data

- Supplementary Data - docx file
