# Supplementary material for: Anemia, Blood Transfusion Requirements and Mortality Risk in Human Immunodeficiency Virus-Infected Adults Requiring Acute Medical Admission to Hospital in South Africa
Source: Open Forum Infect Dis. 2015 Nov 12;2(4):ofv173. doi: 10.1093/ofid/ofv173 (PMC4693115; doi:10.1093/ofid/ofv173)
Supplement: Supplementary Data [file supp_ofv173_ofv173supp_file.docx]

**Supplementary Table 1**. Overview of patients receiving blood transfusion and dying within 90 days following study entry (n=20)

| **Patient** | **Diagnoses** | **HIV status** | **ART status** | **Baseline CD4** | **Baseline**  **viral load (log)** | **Baseline**  **haemoglobin** | **Days until blood transfuion**  **[# units received]** | **Days from study entry to death** |
| --- | --- | --- | --- | --- | --- | --- | --- | --- |
| 1 | 1. New TB (w/mycobacteraemia; previous MDR-TB);  2. Parvovirus B19; | New | Interrupted | 175 | LDL | 3.0 | 0 [2]; 8 [3] | 19 |
| 2 | 1. New TB (w/mycobacteraemia); | New | Naive | 29 | 5.3 | 7.9 | -3 [2] | 85 |
| 3 | 1. AIDS-related (cryprococcal meningitis); 2. Known TB (defaulted treatment) | Known | Interrupted | 47 | 3.1 | 8.7 | 40 [2] | 42 |
| 4 | 1. Major organ dysfunction  (Parvovirus B19)  2. Known TB | Known | Interrupted | 34 | … | 2.7 | 0 [3]; 7 [3] | 37 |
| 5 | 1. New TB (drug-resistant, w/mycobacteraemia)  2. Kidney injury (eGFR=26) | Known | Current | 134 | 3.8 | 8.6 | 27 [1] | 37 |
| 6 | 1. Drug-induced liver injury;  2. Known TB (disseminated);  3. HIV-associated nephropathy (eGFR=40) | Known | Current | 68 | LDL | 6.7 | 1 [3] | 4 |
| 7 | 1. New TB (drug-resistant) | Known | Interrupted | 120 | 6.0 | 9.7 | 11 [1] | 38 |
| 8 | 1. New TB | New | Naive | 23 | 4.6 | 4.4 | -4 [2] | 67 |
| 9 | 1. Bacterial infection (E-coli bacteraemia); 2. Known TB  3. Kidney injury (eGFR=59) | Known | Interrupted | 6 | 3.2 | 5.9 | 14 [2] | 71 |
| 10 | 1. Bacterial infection  (respiratory infection);  2. Known TB | New | Naive | 472 | 4.0 | 6.0 | 1 [2]; 6 [2];  34 [1]; 57 [3] | 60 |
| 11 | 1. Other (gastroenteritis); 2. Kidney impairment(eGFR=34) | Known | Naive | 61 | 6.1 | 4.5 | 1 [2] | 16 |
| 12 | 1. Major organ dysfunction (Parvovirus B19);  2. Kidney injury (eGFR=31) | Known | Current | 9 | 2.7 | 5.7 | -1 [3] | 7 |
| 13 | 1. Known TB (with clinical deterioration);  2. Renal impairment (eGFR=18) | Known | Current | 183 | 2.5 | 3.5 | -1 [2]; 0 [2] | 19 |
| 14 | 1. Drug related (drug-induced liver and kidney injury, eGFR=8);  2. Known TB | Known | Current | 18 | 4.1 | 6.1 | -2 [4]; 1 [2]; 3 [4] | 12 |
| 15 | 1. New TB | Known | Current | 41 | 5.4 | 5.3 | -2 [2] | 79 |
| 16 | Major organ dysfunction (severe renal impairment, eGFR=7) | New | Naive | 131 | 5.7 | 8.2 | 74 [1] | 85 |
| 17 | 1. Bacterial infection (respiratory infection) | New | Naive | … | 6.3 | 4.6 | 0 [2]; 18 [2];  27 [3]; 1 [30] | 32 |
| 18 | 1. Thromboembolism (DVT) | Known | Current | 120 | 4.8 | 8.4 | 14 [2] | 21 |
| 19 | 1. Other (haemangioma)  2. Known TB | Known | Current | 151 | LDL | 8.8 | 28 [2] | 44 |
| 20 | 1. New TB (mycobacteraemia) | New | Naive | 71 | 6.7 | 5.5 | -1 [2] | 5 |

**Abbreviations:** #=number; ART=antiretroviral therapy; DVT= deep venous thrombosis; eGFR=estimated glomerular filtration rate; LDL= lower than detectable limit; TB=tuberculosis; WHO= World Health Organization

**Supplementary Table 2a.** Cox multivariable analysis for risk factors associated with mortality (blood transfusion coded as an ordinal variable)

|  | **Unadjusted HR (95%CI)** | **p-value** | **“Clinical model” Adjusted HR**  **(95% CI)** | **p-value** | **“Classic model” Adjusted HR**  **(95% CI)** | **p-value** |
| --- | --- | --- | --- | --- | --- | --- |
| **Age, for each year increase** | 1.00 (0.98-1.02) | 0.961 |  |  | 1.00 (0.97-1.03) | 0.987 |
| **Male** | 1.02 (0.65-1.60) | 0.936 |  |  | 0.79 (0.46-1.33) | 0.367 |
| **ART status** |  |  |  |  |  |  |
| Current use | 1.0 | 0.919 |  |  |  |  |
| Naïve | 1.02 (0.62-1.69) |  |  |  |  |  |
| Defaulted | 1.13 (0.62-2.05) |  |  |  |  |  |
| **Previous history of TB treatment** |  |  |  |  |  |  |
| No | 1.0 | 0.800 |  |  |  |  |
| Yes | 0.94 (0.60-1.48) |  |  |  |  |  |
| **History of shortness of breath** |  |  |  |  |  |  |
| No | 1.0 | 0.733 |  |  |  |  |
| Yes | 1.08 (0.69-1.69) |  |  |  |  |  |
| **CD4 (cells/μL), for every 50 unit decrease** | 1.10 (1.02-1.19) | 0.004 |  |  | 1.05 (0.97-1.14) | 0.230 |
| **Viral load (copies/mL), for each log unit increase** | 0.99 (0.87-1.13) | 0.871 |  |  |  |  |
| **Haemoglobin (g/dL), for each unit decrease** | 1.15 (1.06-1.25) | 0.001 | 1.10 (1.00-1.21) | 0.057 | 1.10 (0.99-1.23) | 0.073 |
| **CRP (mg/L), for each 10 unit increase** | 1.03 (1.01-1.06) | 0.007 |  |  | 1.03 (1.00-1.06) | 0.058 |
| **eGFR category (mL/min/1.73 m^2^)** |  |  |  |  |  |  |
| eGFR ≥30 | 1.0 | <0.001 | 1.0 | **<0.001** | 1.0 | **0.005** |
| eGFR<30 | 3.72 (2.08-6.64) |  | 3.25 (1.76-6.02) |  | 2.81 (1.43-5.52) |  |
| **Confirmed mycobacteraemia** |  |  |  |  |  |  |
| No | 1.0 | 0.103 |  |  | 1.0 | 0.135 |
| Yes | 1.80 (0.93-3.53) |  |  |  | 2.06 (0.82-5.19) |  |
| **Clinical Diagnosis** |  |  |  |  |  |  |
| “Other” | 1.0 | 0.002 |  |  | 1.0 | **<0.001** |
| New TB | 1.10(0.59-2.05) |  |  |  | 0.70 (0.31-1.58) |  |
| Deterioration of TB | 1.00 (0.34-2.94) |  |  |  | 1.07 (0.34-3.34) |  |
| AIDS-defining illness (other than TB) | 3.40 (1.80-6.43) |  |  |  | 3.41 (1.62-7.18) |  |
| NCD/MOD | 1.84 (0.92-3.67) |  |  |  | 2.38 (1.08-5.24) |  |
| **Cardiopulmonary illness** |  |  |  |  |  |  |
| Yes | 1.0 | 0.050 | 1.0 | **0.028** |  |  |
| No | 1.57 (1.00-2.49) |  | 1.67 (1.05-2.66) |  |  |  |
| **Blood transfusion category** |  |  |  |  |  |  |
| 0 units received | 1.0 | 0.018 | 1.0 | 0.390 | 1.0 | 0.501 |
| 1-3 units received | 2.20 (1.23-3.95) |  | 1.52 (0.79-2.91) |  | 1.12 (0.52-2.40) |  |
| 4-7 units received | 2.02 (0.63-6.43) |  | 0.69 (0.19-2.55) |  | 0.45 (0.09-2.23) |  |
| ≥8 units received | 4.13 (1.29-13.19) |  | 1.77 (0.51-6.10) |  | 1.74 (0.50-6.08) |  |

**Abbreviations:** ART=antiretroviral therapy; CRP=C-reactive protein; eGFR=estimated glomerular filtration rate; HR=hazard ratio; MOD=major organ dysfunction; NCD= non-communicable disease; TB=tuberculosis

|  | **Unadjusted HR (95%CI)** | **p-value** | **“Clinical model” Adjusted HR**  **(95% CI)** | **p-value** | **“Classic model” Adjusted HR**  **(95% CI)** | **p-value** |
| --- | --- | --- | --- | --- | --- | --- |
| **Age, for each year increase** | 1.00 (0.98-1.02) | 0.961 |  |  | 1.00 (0.98-1.03) | 0.844 |
| **Male** | 1.02 (0.65-1.60) | 0.936 |  |  | 0.80 (0.48-1.35) | 0.402 |
| **ART status** |  |  |  |  |  |  |
| Current use | 1.0 | 0.919 |  |  |  |  |
| Naïve | 1.02 (0.62-1.69) |  |  |  |  |  |
| Defaulted | 1.13 (0.62-2.05) |  |  |  |  |  |
| **Previous history of TB treatment** |  |  |  |  |  |  |
| No | 1.0 | 0.800 |  |  |  |  |
| Yes | 0.94 (0.60-1.48) |  |  |  |  |  |
| **History of shortness of breath** |  |  |  |  |  |  |
| No | 1.0 | 0.733 |  |  |  |  |
| Yes | 1.08 (0.69-1.69) |  |  |  |  |  |
| **CD4 (cells/μL), for every 50 unit decrease** | 1.10 (1.02-1.19) | 0.004 |  |  | 1.06 (0.97-1.15) | 0.185 |
| **Viral load (copies/mL), for each log unit increase** | 0.99 (0.87-1.13) | 0.871 |  |  |  |  |
| **Haemoglobin (g/dL), for each unit decrease** | 1.15 (1.06-1.25) | 0.001 | 1.10 (1.00-1.21) | **0.038** | 1.09 (0.98-1.22) | 0.102 |
| **CRP (mg/L), for each 10 unit increase** | 1.03 (1.01-1.06) | 0.007 |  |  | 1.03 (1.00-1.06 | **0.050** |
| **eGFR category (mL/min/1.73 m^2^)** |  |  |  |  |  |  |
| eGFR ≥30 | 1.0 | <0.001 | 1.0 | **0.001** | 1.0 | **0.010** |
| eGFR<30 | 3.72 (2.08-6.64) |  | 3.09 (1.67-5.73) |  | 2.53 (1.30-4.94) |  |
| **Confirmed mycobacteraemia** |  |  |  |  |  |  |
| No | 1.0 | 0.103 |  |  | 1.0 | 0.142 |
| Yes | 1.80 (0.93-3.53) |  |  |  | 1.99 (0.81-4.91) |  |
| **Clinical Diagnosis** |  |  |  |  |  |  |
| “Other” | 1.0 | 0.002 |  |  | 1.0 | **<0.001** |
| New TB | 1.10(0.59-2.05) |  |  |  | 0.68 (0.30-1.53) |  |
| Deterioration of TB | 1.00 (0.34-2.94) |  |  |  | 1.03 (0.33-3.21) |  |
| AIDS-defining illness (other than TB) | 3.40 (1.80-6.43) |  |  |  | 3.39 (1.62-7.13) |  |
| NCD/MOD | 1.84 (0.92-3.67) |  |  |  | 2.34 (1.06-5.15) |  |
| **Cardiopulmonary illness** |  |  |  |  |  |  |
| Yes | 1.0 | 0.050 | 1.0 | **0.028** |  |  |
| No | 1.57 (1.00-2.49) |  | 1.67 (1.05-2.66) |  |  |  |
| **Blood transfusion, for each 1 unit received** | 1.10 (1.02-1.18) | 0.048 | 1.03 (0.90-1.17) | 0.699 | 1.02 (0.88-1.18) | 0.810 |

**Supplementary Table 2b.** Cox multivariable analysis for risk factors associated with mortality (blood transfusion coded as a continuous variable)

lklk

**Abbreviations:** ART=antiretroviral therapy; CRP=C-reactive protein; eGFR=estimated glomerular filtration rate; HR=hazard ratio; MOD=major organ dysfunction; NCD= non-communicable disease; TB=tuberculosis

|  | **Received transfusion**  **n=63** | **No transfusion**  **n=114** | **p-value** |
| --- | --- | --- | --- |
| **Patient characteristics** |  |  |  |
| Age, median (IQR) | 32.2 (25.7-38.7) | 33.0 (28.0-39.0) | 0.235 |
| Female | 43 (68.3) | 62 (54.4) | 0.072 |
| HIV newly diagnosed | 16 (25.4) | 25 (21.9) | 0.710 |
| **ART status** |  |  |  |
| ART-naïve | 27 (42.9) | 50 (43.9) | 0.519 |
| Current ART use | 26 (41.3) | 39 (34.2) |  |
| ART interrupted | 10 (15.9) | 25 (21.9) |  |
| Receiving AZT-containing regimen if currently receiving ART^a^ | 4 (6.4) | 2 (1.8) | 0.188 |
| If currently on ART, treatment duration (years), median (IQR)^a^ | 0.5 (0.2-2.2) | 1.3 (0.2-2.6) | 0.692 |
| If currently on ART, proportion taking ART for <90 days^a^ | 8 (30.8) | 13 (34.1) | 0.773 |
| **Haemoglobin (g/dL),** median (IQR) | 5.5 (4.6-6.5) | 7.1 (6.4-7.6) | <0.001 |
| **WHO Anaemia severity** |  |  |  |
| Severe | 16 (25.4) | 84 (73.7) | <0.001 |
| Life-threatening | 47 (74.6) | 30 (26.3) |  |
| **CD4 cell count (cells/μL)** |  |  |  |
| Median (IQR) | 71 (32-190) | 72 (32-186) | 0.741 |
| **Log viral load (copies/ml),** median (IQR)^b^ | 4.0 (2.3-5.3) | 5.4 (3.1-5.9) | 0.005 |
| **Viral load<400 (VL suppression)** | 20 (32.3) | 20 (19.1) | 0.053 |
| **C-reactive protein (mg/L)^c^** | 91 (45-166) | 99 (54-159) | 0.661 |
| **eGFR classification** (mL/min/1.73 m^2^) |  |  |  |
| <30 | 12 (19.1) | 13 (11.4) | 0.162 |
| ≥30 | 51 (81.0) | 101 (88.6) |  |
| **Symptoms** |  |  |  |
| Short of breath (self-reported) | 28 (45.2) | 52 (46.0) | 1 |
| Cough (current, self-reported) | 26 (41.3) | 56 (49.6) | 0.291 |
| **Tuberculosis** |  |  |  |
| History of previous TB | 19 (30.2) | 59 (51.8) | 0.006 |
| Positive WHO symptom screen | 60 (95.2) | 112 (98.3) | 0.248 |
| Mycobacteraemia^d^ | 15 (25.0) | 14 (12.6) | 0.039 |
| Cardiopulmonary illness present | 30 (47.6) | 66 (57.9) | 0.189 |
| **Clinical Diagnosis** |  |  |  |
| New TB | 25 (41.7) | 53 (48.2) | 0.004 |
| Deterioration of TB | 5 (8.3) | 9 (8.2) |  |
| AIDS-defining illness (other than TB) | 1 (1.7) | 12 (10.9) |  |
| NCD/MOD | 17 (28.3) | 9 (8.2) |  |
| Other | 20 (12) | 27 (24.6) |  |

**Supplementary Table 3.** Characteristics stratified according to blood transfusion status within 90 days months following study entry among patients with severe anaemia or life-threatening anaemia (n=177)

**Abbreviations:** ART=antiretroviral therapy; AZT=zidovudine; CRP=C-reactive protein; eGFR=estimated glomerular filtration rate; MOD=major organ dysfunction; NCD= non-communicable disease TB=tuberculosis; WHO= World Health Organization

All values are numbers (%) unless otherwise stated.

^a^n=260,^b^n=576,^c^n=562, ^d^n=554,^e^n=552

**Supplementary Table 4.** Multivariable logistic regression of factors associated with receipt of blood transfusion within 90 days following study entry among patients with severe or life-threatening anaemia (n=177)

|  | **Unadjusted OR (95%CI)** | **p-value** | **Model 1 Adjusted OR** | **p-value** | **Model 2 Adjusted OR** | **p-value** |
| --- | --- | --- | --- | --- | --- | --- |
| **Age** | 0.98 (0.94-1.02) | 0.351 |  |  |  |  |
| **Female** | 1.80 (0.95-3.44) | 0.070 | 2.23 (0.95-5.21) | 0.058 | 2.06 (0.88-4.80) | 0.089 |
| **ART status** |  |  |  |  |  |  |
| Current | 1.0 | 0.514 |  |  |  |  |
| Naïve | 0.81 (0.41-1.60) |  |  |  |  |  |
| Defaulted | 0.60 (0.25-1.45) |  |  |  |  |  |
| **History of TB** |  |  |  |  |  |  |
| Yes | 1.0 | 0.005 | 1.0 | 0.126 | 1.0 | 0.121 |
| No | 2.48 (1.29-4.77) |  | 1.91 (0.93-4.39) |  | 1.91 (0.94-4.36) |  |
| **History of shortness of breath** | 0.97 (0.52-1.80) | 0.913 |  |  |  |  |
| **CD4 (cells/μL), for each 50 unit decrease** | 1.05 (0.95-1.16) | 0.366 |  |  |  |  |
| **HIV viral load (copies/mL), for each log unit increase** | 1.28 (1.06-1.53) | 0.008 | 1.14 (0.89-1.45) | 0.300 | 1.24 (0.97-1.58) | 0.078 |
| **Haemoglobin (g/dL), for each unit decrease** | 3.04 (2.13-4.33) | <0.001 | 2.83 (1.92-4.15) | <0.001 | 2.88 (1.97-4.22) | <0.001 |
| **eGFR** **(mL/min/1.73 m^2^)** |  |  |  |  |  |  |
| ≥30 | 1.0 | 0.169 |  |  |  |  |
| <30 | 1.83 (0.78-4.29) |  |  |  |  |  |
| **CRP (mg/L), for each 10 unit increase** | 1.00 (0.96-1.04) | 0.981 |  |  |  |  |
| **Mycobacterial blood culture** |  |  |  |  |  |  |
| No | 1.0 | 0.043 |  |  | 1.0 |  |
| Yes | 2.31 (1.03-5.19) |  |  |  | 3.03 (0.97-9.52) | 0.056 |
| **Clinical Diagnosis** |  |  |  |  |  |  |
| Other | 1.0 | 0.005 | 1 | 0.682 |  |  |
| New TB | 0.98 (0.44-2.20) |  | 1.11 (0.41-3.00) |  |  |  |
| Deterioration of TB | 1.20 (0.33-4.29) |  | 1.23 (0.25-6.51) |  |  |  |
| AIDS-defining illness (other than TB) | 0.36 (0.07-1.84) |  | 0.60 (0.09-3.96) |  |  |  |
| NCD/MOD | 4.31 (1.53-12.14) |  | 2.28 (0.61-8.44) |  |  |  |
| **Cardiopulmonary illness (not including TB)*** |  |  |  |  |  |  |
| No | 1.0 | 0.189 |  |  | 1.0 | 0.792 |
| Yes | 1.51 (0.81-2.81) |  |  |  | 1.12 (0.47-2.68) |  |

**Abbreviations:** ART=antiretroviral therapy; CRP=C-reactive protein; eGFR=estimated glomerular filtration rate; MOD=major organ dysfunction; NCD= non-communicable disease; OR=odds ratio; TB=tuberculosis

*pre-specified risk factor
